# Gender and Age-Dependent Etiology of Community-Acquired Urinary Tract Infections

**DOI:** 10.1100/2012/349597

**Published:** 2012-04-26

**Authors:** Enrico Magliano, Vittorio Grazioli, Loredana Deflorio, Antonia Isabella Leuci, Roberto Mattina, Paolo Romano, Clementina Elvezia Cocuzza

**Affiliations:** ^1^Bacteriological Laboratory, Centro Diagnostico Italiano, Via Saint Bon 20, 20147 Milan, Italy; ^2^Department of Public Health, Microbiology and Virology, University of Milan, Via Pascal 36, 20166 Milan, Italy; ^3^Department of Clinical and Preventive Medicine, University of Milano-Bicocca, Via Cadore 48, 20052 Monza, Italy

## Abstract

Urinary tract infections (UTIs) are among the most frequent community-acquired infections worldwide. *Escherichia coli* is the most common UTI pathogen although underlying host factors such as patients' age and gender may influence prevalence of causative agents. In this study, 61 273 consecutive urine samples received over a 22-month period from outpatients clinics of an urban area of north Italy underwent microbiological culture with subsequent bacterial identification and antimicrobial susceptibility testing of positive samples. A total of 13 820 uropathogens were isolated and their prevalence analyzed according to patient's gender and age group. Overall *Escherichia coli* accounted for 67.6% of all isolates, followed by *Klebsiella pneumoniae* (8.8%), *Enterococcus faecalis* (6.3%), *Proteus mirabilis* (5.2%), and *Pseudomonas aeruginosa* (2.5%). Data stratification according to both age and gender showed *E. coli* isolation rates to be lower in both males aged ≥60 years (52.2%), *E. faecalis* and *P. aeruginosa* being more prevalent in this group (11.6% and 7.8%, resp.), as well as in those aged ≤14 years (51.3%) in whom *P. mirabilis* prevalence was found to be as high as 21.2%. *Streptococcus agalactiae* overall prevalence was found to be 2.3% although it was shown to occur most frequently in women aged between 15 and 59 years (4.1%). Susceptibility of *E. coli* to oral antimicrobial agents was demonstrated to be as follows: fosfomycin (72.9%), trimethoprim/sulfamethoxazole (72.9%), ciprofloxacin (76.8%), ampicillin (48.0%), and amoxicillin/clavulanate (77.5%). In conclusion, both patients' age and gender are significant factors in determining UTIs etiology; they can increase accuracy in defining the causative uropathogen as well as providing useful guidance to empiric treatment.

## 1. Background

Urinary tract infections (UTIs) are among the most frequent bacterial infections worldwide [1–5]. In 2000, according to the Urologic Diseases in America Project, UTIs accounted for more than 8 million office and 1,7 million emergency room visits, leading to around 350 000 hospitalizations [3]. In England and Wales, consulting rates in general practice for cystitis and other urinary infections were found to be of approximately 315 per 10 000 persons [4], whereas, in Italy, in 2002, 2.4% of a cohort of more than 450 000 people received a diagnosis of acute cystitis in primary care [5]. 

Although women, particularly those aged 16–64 years, are significantly more likely to experience UTIs than men [4], urinary infections frequently occur in both genders and across all age groups [3, 4]; specific populations such as pregnant women, the elderly or patients with spinal cord injuries, catheters, or diabetes are also at increased risk [6, 7]. 

Microbial etiology of UTIs has been regarded as well established, with *E. coli* being the causative pathogen in 50–80% of cases [8, 9]; other Enterobacteriaceae (*Klebsiella, Proteus, Enterobacter*) together with Enterococci, Streptococci, Staphylococci, and *Pseudomonas spp*. account for most of the remaining positive urine cultures [8]. Empiric antibiotic treatment is therefore commonly adopted. However, due to significant local differences in frequency of urinary agents, the emergence of new pathogens, and changes of antimicrobial resistance, periodic evaluation of pathogens epidemiology is recommended, in order to revise treatment advices [10]. Since underlying host factors may affect urinary etiology and antibiotic susceptibility, specific patients groups should be investigated in more details. Among risk factors, patients' gender and age can be easily accessible in surveys performed at the microbiology laboratory level where patients' clinical features are less well known. The present study was therefore conducted with the aim to assess UTI etiology and antimicrobial susceptibility of a large number of urinary pathogens isolated in an urban area of north of Italy as well as to evaluate bacteria distribution according to age and gender.

## 2. Methods

A retrospective study was performed at the Bacteriological Laboratory of the “Centro Diagnostico Italiano” (CDI), based in Milan (Italy), on all bacterial strains isolated from consecutive urine samples received from outpatients clinics of a high-populated urban area of North Italy, between March 2008 and December 2009. Urine samples, accompanied by microbiology request forms, were delivered either directly to the CDI laboratory or through 7 collaborating laboratories. All sample processing and patients' data collection were carried out centrally by the CDI laboratory.

CDI Laboratory follows Internal Quality Control procedures and participates to an External Program for Quality Assessment with positive evaluations. 

As part of the routine procedure, patients received indications to avoid antimicrobials assumption during the previous 7 days and instructions on urine sampling (including cleaning of the genital area prior to midstream specimen collection) and its transport to the laboratories (within 2 hours of collection). Specimens from collaborating laboratories were transported in Vacutainer tubes containing boric acid at 1-2% as preservative. All samples were plated as soon as possible and no later than 24 hours on Chromagar Orientation-BD plates and incubated for 18–24 hours at 37°C. Criterion for defining significant bacteriuria (positive samples) was the presence of ≥10^5^ colony-forming units (CFU)/mL of urine [11]. 

An automated BD Phoenix system (BD Biosciences, USA) was used for the rapid bacteria identification and for the determination of antimicrobial susceptibility [12]. Phoneix AST antimicrobials panels for Gram-negative urinary pathogens, Gram-negative nonurinary pathogens, *Streptococci,* and all other Gram-positive were used.

The statistical analysis was performed by the SAS System version 9.2. The difference between females and males in the frequency of positive samples of each agent was analyzed by the chi-square test. The frequency of the positive samples of each agent as a function of gender and age groups (≤14; 15–29; 30–59; ≥60 years) was analyzed by the logistic regression, including in the model the terms gender, age, and the gender by age interaction.

## 3. Results

A total of 61 273 urine cultures were performed over a 22-month period; of these 13 820 (22.6%) were found to be positive for bacterial infection. Nearly 80% of all isolates were from women (female to male ratio (F/M) = 3.8) and 58% from subjects aged 60 years or more. Cumulatively, the two younger age groups accounted for 11.2% of total isolates. Female to male ratio was highest in age group 15–29 years (F/M = 13.5) and lowest in the youngest age group (F/M = 1.4).

Overall the most frequently encountered pathogen was *Escherichia coli* (67.6%), followed by *Klebsiella pneumoniae* (8.8%), *Enterococcus faecalis* (6.3%), *Proteus mirabilis* (5.2%), *Pseudomonas aeruginosa* (2.5%), and *Streptococcus agalactiae* (2.3%) (Table 1), all accounting for around 90% of total isolates. Gram-negative agents represented 90.8% of urinary pathogens.

Frequency of isolation of all six main species was found to be statistically different between females and males (Table 2): *E. coli*, *K. pneumonia,* and *S. agalactiae* were more frequent in females, whereas *E. faecalis*, *P. mirabilis,* and *P. aeruginosa* were more common in men. 

All the six most prevalent bacterial species, with the exception of *K. pneumoniae,* revealed statistically significant differences in isolation rates within the four chosen age groups (Table 3) with *E. coli* being less prevalent in the youngest subjects (58.9%) and more frequent in the age groups 15–29 (71.0%) and 30–59 years (71.0%). 

Data stratification according to both gender and age showed that differences in frequency of isolation between females and males of *E. faecalis* and *S. agalactiae* were not consistent across all age groups (Table 3). Furthermore, *E. coli* isolation rates were shown to be lower in males aged ≥60 years (52.2%), whilst *E. faecalis* and *P. aeruginosa* were shown to be more prevalent in this group (11.6% and 7.8%, resp.), and in those aged ≤14 years (51.3%). Interestingly *P. mirabilis* prevalence was found to be highest (21.2%) in young males, aged ≤14 years.

Susceptibility to antimicrobials of main isolated uropathogens is shown in Table 4. *E. coli* susceptibility to orally active compounds ranged from 48.0% (ampicillin) to 97.0% (fosfomycin): susceptibility to trimethoprim-sulfamethoxazole (TMP-SMX) was 72.9%. Tested quinolones compounds resulted to be equally active against uropathogenic *E. coli* (ranging from 76.6% to 77.1%), and amoxicillin/clavulanate rate of activity (77.5%) was comparable to that of quinolones. Piperacillin was the parenteral antibiotic with the lowest rate of activity (51.9%) against *E. coli*; susceptibility of *E. coli* to gentamicin, ceftriaxone, and cefazolin ranged from 84.3% to 91%, while rates for piperacillin/tazobactam, amikacin, meropenem, and imipenem were higher (from 95.4% to 100%). 

*K. pneumoniae* susceptibility to quinolones (93.0%–95.3%) and to TMP-SMX (89.8%) was higher in comparison to *E. coli* while fosfomycin activity resulted to be lower (81.0%). *E. faecalis* susceptibility to ampicillin and fosfomycin was high (96.1% and 100%, resp.), superior with respect to susceptibility to quinolones (71.9%–82.3%).

*S. agalactiae* susceptibility to levofloxacin was found to be 91.1% (data not shown).

## 4. Discussion

As urinary tract infection is a very common disease, its diagnosis and treatment have important implications for patients' health, development of antibiotic resistance, and health care costs [1–5]. Surveillance of local UTI's etiology as well as of antimicrobial susceptibility is considered useful to guide empirical therapy, as prevalence of pathogens and their features may vary with time and geographical area [10]. Attempts should also be made to increase prediction of causative uropathogens through the use of demographic and clinical available data. 

The present retrospective study describes the distribution and antimicrobial susceptibility of bacterial species isolated from a large number of urinary samples collected over a 22-month period, as part of routine analyses, from unselected community patients (male and female of any age and clinical condition) living in a urban area in the north of Italy. The high number of available isolates allowed to stratify data according to patients' gender and age and so to evaluate the association of such variables to UTI etiology.

As expected *E. coli* was the most frequently encountered species in our study. Percentage of *E. coli* isolation (67.6%) well compares with those reported from other outpatients surveys conducted in north (64.6%) [13] and south (68.0%) [14] of Italy. Other investigations conducted in Europe [15–19] found percentages of *E. coli* isolation ranging from 47.6% [16] to 85.9% [19], while, in North and Latin America, figures from 57.5% to 71.6% were reported [20, 21]. Unfortunately, true differences in local etiology are often difficult to establish due to large differences in study design, number of samples, and patients' entry criteria as well as in data presentation, among different reports.

Other frequent isolates found in this study included *K. pneumoniae*, *P. mirabilis,* and *E. faecalis*, all having been reported to be highly prevalent species in UTIs [15, 16, 20], and *P. aeruginosa* and *S. agalactiae* whose frequency of isolation seems to be less constant across surveys. Isolation rates of Gram-positive bacteria other than *E. faecalis* and *S. agalactiae* were only 0.7%; *S. aureus* was responsible for 0.4% of these, a similar finding was observed by De Francesco et al. in north of Italy (0.9%) in year 2005 [13]. 

Our study, however, showed that prevalence of urinary pathogens following data stratification was not consistent across all age groups further divided by gender. *Escherichia coli,* for example, was found to be less prevalent in the youngest and oldest male subjects (51.3% and 52.2%, resp.) and more frequent in female patients aged 15 years or older (approximately 71%), *Proteus mirabilis* prevalence was found to be highest (21.2%) in young males aged ≤14 years, whilst *S. agalactiae *was mostly found in women aged between 15 and 59 years (approximately 4.0%).

Kiffer et al. [21] conducted a study comparable to ours, in terms of patient's population (both males and females of any age), number of isolates (35 782), and selected age groups. They also found (1) a lower percentage of *E. coli* isolation in patients younger than 13 years or older than 60 years (69.0% and 68.8%, resp.) as compared to the age group 13–60 years (79.7%); (2) a higher difference in *E. coli* rates of isolation, between males and females, in the youngest (27.2%) and the oldest (25.8%) age groups with respect to the 13–60 years age group (8.9%); (3) a higher prevalence of *E. faecalis* (16.4%) and *P. aeruginosa* (14.7%) in males older than 60 years, approximately three and six times higher, respectively, as compared to females of the same age group; (4) *P. mirabilis* to be the second leading cause of UTI in pediatric population (0–13 years), accounting for 22.1% of isolates (15.6% in our study) with a females to males ratio of 0.45 (0.55 in our study). 

Already in 1972, Bergström [22] and, more recently, Modarres and Oskoii [23] reported *P. mirabilis* to be a common cause of urinary tract infections in boys, although this finding has not been confirmed by other studies on children UTIs [24, 25].

*P. mirabilis* has been described to be present in the preputial sac of boys, having been isolated in 22.6% of uncircumcised males of up to 14 years of age [26]; this can support the role of *P. mirabilis* as an important urinary pathogen in this group of patients. However, *P. mirabilis* also seems to represent an important urinary pathogen in young females as supported by the high isolation rates in this group of patients observed in our study and by Kiffer et al. [21], in spite of its low prevalence in the preadolescent female genital tract flora [27].

Susceptibility of uropathogenic bacteria to antimicrobials agents is also known to vary among countries and over time [10]. European Urology Association (EUA) Guidelines [6] recommend trimethoprim/sulfamethoxazole (TMP/SMX) as first line drug for empirical therapy in community-acquired infections, when local rates of resistance of uropathogens to TMP are <10–20%.

In our survey, 72.9% of *E. coli* isolates were susceptible to TMP/SMX. Comparable figures of *E. coli* susceptibility to TMP/SMX were found in Italy by De Francesco et al. (range of 70%–72% between 2002 and 2005) [13] and Miragliotta et al. (71.6% overall, from 2001 to 2006) [14]. Although values may vary among reports, resistance rate of recently community isolated of *E. coli* to TMP/SMX in Europe tends to be >20% [16], having also being reported higher than 30% [28, 29]. *E. coli* susceptibility to fluoroquinolones in our study ranged from 76.6% (norfloxacin) to 77.1% (levofloxacin) which was similar to rates observed in Italy by De Francesco et al. (78% and 80%, resp.) [13] and Miragliotta et al. (78.7% and 79.6%) [14]. *E. coli* susceptibility to ampicillin in our study was found to be low (48%) and comparable to other reports from Italy (51.0% and 44.0%) [13, 14] and Europe [8, 16]. Susceptibility of *E. coli* to amoxicillin/clavulanate in our study was higher (77.5%) than that observed for ampicillin and similar to what recently reported from Italy by Miragliotta et al. (73.1%) [14] and Schito et al. (71.5%) [16], although De Francesco et al. found even higher susceptibility rates (90%) [13] more in line with other recent reports from Europe [16, 19]. Among the oral antimicrobial compounds tested in our study fosfomycin exhibited the highest activity against *E. coli* (97.0%). A comparable result was shown in a number of other studies conducted in Italy and Europe [8, 14, 16, 30] where *E. coli* susceptibility to fosfomycin ranged between 90.8% [14] and 98.1% [16].

Susceptibility to oral antimicrobials of *P. mirabilis* strains isolated in our study, was generally lower than that reported both in Italy and other countries. Susceptibility to ciprofloxacin and TMP/SMX of *P. mirabilis* isolates in our study was of 62.9% and 51.5%, respectively, as compared to rates demonstrated by other authors ranging from 75.5% to 97.9% for ciprofloxacin and from 52.0% to 84.9% for TMP-SMX [8, 13–16]. Similarly, rates of *P. mirabilis* susceptibility to ampicillin, amoxicillin/clavulanate, and fosfomycin were lower in this study (38.9%, 67.0%, and 68.8%, resp.), in comparison to those described in previous reports where ranges of susceptibility rates of 53.6%–83.9% to ampicillin, 79.6%–99.0% to amoxicillin/clavulanate, and 60.0%–96.9% to fosfomycin were reported [8, 13–16].

In conclusion, besides providing further data on the etiology of community-acquired UTIs and antimicrobial susceptibility of uropathogens in Italy, our results confirm that stratification of isolates from unselected patients on the basis of age and gender can improve the assessment of causative pathogens, providing guidance for empiric treatment and interesting clues to the understanding of UTIs etiopathology. 

In particular,* P. mirabilis* prevalence was found to be high both in boys (21.2%) and girls (11.8%) suggesting, as previously reported [30], that fosfomycin could represent a drug of choice for the therapy of children's UTIs, especially when considering its good antibacterial activity against both *E. coli* and *P. mirabilis* (higher as compared to *β*-lactams) and the concerns on fluoroquinolones use in children. For other patients' subgroups, it was noted that frequently isolated bacterial species, such as *E. faecalis* or *P. aeruginosa* in older males, showed different antimicrobial susceptibilities as compared to *E. coli*, underlying the importance that empiric treatment should be based on epidemiological data which takes into account patients gender and age.

## Figures and Tables

**Table 1:** Distribution of bacterial isolates from urine samples (*n* = 13820). Data are reported as number of isolates and percentages (within brackets) of total.

Gram-negative		Gram-positive	
*E. coli *	9344 (67.6)	*Enterococcus spp.* ^g^	879 (6.4)
*K. pneumoniae* ^a^	1217 (8.8)	*S. agalactiae*	313 (2.3)
*Proteus spp.* ^b^	734 (5.3)	*S. aureus*	51 (0.4)
*P. aeruginosa *	345 (2.5)	*Staphylococcus spp.* ^h^	28 (0.2)
*Enterobacter spp.* ^c^	295 (2.1)		
*K. oxytoca*	239 (1.7)		
*Citrobacter spp.* ^d^	227 (1.6)		
*M. morganii*	83 (0.6)		
*Providencia spp.* ^e^	46 (0.3)		
*Other Gram-negative* ^f^	19 (0.1)		

*All Gram-negative*	12549 (90.8)	*All Gram-positive*	1271 (9.2)

Includes: ^**a**^ *ssp pneumoniae (1204 isolates) *and *ozaenae (13);* ^b**^ *P. mirabilis (723) *and *P. vulgaris (11);* ^c**^ *E. aerogenes (153) *and *E. cloacae (142);* ^d**^ *C. koseri (180), C. freundii (42), C. braakii (3), *and *C. youngae (2);* ^e**^ *P. stuartii (38) *and *P. rettgeri (8);* ^**f**^ *A. baumannii (9), S. marcescens (8), *and *S. maltophilia (2);* ^g**^ *E. faecalis (868) *and *E. faecium (11);* ^h**^ *S. saprophyticus (21), S. epidermidis (4), S. haemolyticus (2), *and *S. warneri (1).*

**Table 2:** Distribution of the six more frequently isolated species according to patients' gender.

Organism	All	Females	Males	
	(*n* = 13820)	(*n* = 10947)	(*n* = 2873)	*P* ^ a^
*E. coli *	9344 (67.6)	7763 (71.0)	1581 (55.0)	<0.001
*K. pneumonia *	1217 (8.8)	995 (9.1)	209 (7.3)	0.002
*E. faecalis*	868 (6.3)	596 (5.4)	272 (9.5)	<0.001
*P. mirabilis*	723 (5.2)	465 (4.2)	258 (9.0)	<0.001
*P. aeruginosa*	345 (2.5)	149 (1.4)	196 (6.8)	<0.001
*S. agalactiae*	313 (2.3)	270 (2.5)	43 (1.5)	0.002
*All other G−*	933 (6.8)	651 (5.9)	282 (9.8)	<0.001
*All other G+*	90 (0.7)	58 (0.5)	32 (1.1)	0.001

^a^ *P*  value (chi-square) of the comparison between females and males.

**Table 3:** Distribution of urine pathogens according to age groups and gender. Data are reported as number of isolates and percentages (within brackets) of total patients in each age group.

Organism		Age groups				
	Gender	≤14 years	15–29 years	30–59 years	≥60 years	*P*
	All	703^a^	841	4167	8109	
	Females	415	783	3615	6134	
	Males	288	58	552	1975	
*E. coli *	All	414 (58.9)	597 (71.0)	2959 (71.0)	5374 (66.3)	0.0005^b^
	Female	266 (64.1)	562 (71.8)	2591 (71.7)	4344 (70.8)	< 0.0001^c^
	Male	148 (51.3)	35 (60.3)	368 (66.7)	1030 (52.2)	
*K. pneumoniae*	All	43 (6.1)	77 (9.2)	369 (8.6)	715 (8.8)	0.4244
	Female	26 (6.3)	71 (9.1)	325 (9.0)	573 (9.3)	0.5626
	Male	17 (5.9)	6 (10.3)	44 (8.0)	142 (7.2)	
*E. faecalis*	All	18 (2.6)	45 (5.4)	258 (6.2)	547 (6.7)	0.0004
	Female	14 (3.4)	42 (5.4)	222 (6.1)	318 (5.2)	0.9935
	Male	4 (1.4)	3 (5.2)	36 (6.5)	229 (11.6)	
*P. mirabilis *	All	110 (15.6)	31 (3.7)	160 (3.8)	422 (5.2)	0.0055
	Female	49 (11.8)	24 (3.1)	128 (3.5)	264 (4.3)	<0.0001
	Male	61 (21.2)	7 (12.1)	32 (5.8)	158 (8.0)	
*P. aeruginosa*	All	40 (5.7)	10 (1.2)	37 (0.9)	258 (3.2)	<0.0001
	Female	21(5.1)	6 (0.8)	19 (0.5)	103 (1.7)	<0.0001
	Male	19 (6.6)	4 (6.9)	18 (3.3)	155 (7.8)	
*S. agalactiae *	All	2 (0.3)	31 (3.7)	157 (3.8)	123 (1.5)	<0.0001
	Female	1 (0.2)	31 (4.0)	150 (4.1)	88 (1.4)	0.0939
	Male	1 (0.3)	0 (0)	7 (1.3)	35 (1.8)	

^a^patients' numbers; logistic regression analysis among age groups of distribution of isolation rates in ^b^All patients or ^c^by Gender.

**Table 4:** Susceptibility rates to oral and parenteral antimicrobials of most common uropathogens isolated from urine samples.

Organism (n; %)	Antimicrobials															
	CIP	LVX	NOR	NIT	SXT	AMP	AMC	FOS	PIP	TZP	CFZ	CRO	MRP	IMP	GEN	AMK
*E. coli (9344;67.6%)*	76.8	77.1	76.6	95.1	72.9	48.0	77.5	97.0	51.9	95.4	84.3	90.0	100	99.9	91.0	99.6
*K. pneumonia (1204;8.7%)*	93.6	93.0	95.3	35.1	89.8	0	81.4	81.0	61.8	92.3	78.3	87.9	99.7	99.9	92.9	97.1
*E. faecalis (868;8.3%)*	71.9	82.3	—	96.1	—	96.1	—	100.0	—	—	—	—	—	—	—	—
*P. mirabilis (723;5.2%)*	62.9	75.2	78.3	0	51.5	38.9	67.0	60.8	64.8	98.9	65.4	79.0	100	94.0	67.7	99.3
*P. aeruginosa (345;2.3%)*	57.7	58.7	60.6	0	0	0	0	0	88.4	95.9	0	0	94.7	93.3	68.0	83.7

—: not tested; CIP: ciprofloxacin; LVX: levofloxacin; NOR: norfloxacin; NIT: nitrofurantoin; SXT: trimethoprim-sulfamethoxazole; AMP: ampicillin; AMC: amoxicillin/clavulanic acid; FOS: fosfomycin; PIP: piperacillin; TZP: piperacillin/tazobactam; CFZ: cefazolin; CRO: ceftriaxone; MRP: meropenem; IMP: imipenem; GEN: gentamicin; AMK: amikacin.

## References

[1] Ronald AR, Nicolle LE, Stamm E (2001). Urinary tract infection in adults: research priorities and strategies. *International Journal of Antimicrobial Agents*.

[2] Foxman B, Barlow R, D’Arcy H, Gillespie B, Sobel JD (2000). Urinary tract infection: self-reported incidence and associated costs. *Annals of Epidemiology*.

[3] Litwin MS, Saigal CS, Yano EM (2005). Urologic diseases in America project: analytical methods and principal findings. *Journal of Urology*.

[4] Royal College of General Practitioners, Office of Population Censuses and Surveys, Department of Health (1995 ). *Morbidity Statistics from General Practice: Fourth National Study 1991-1992*.

[5] Galatti L, Sessa A, Mazzaglia G (2006). Antibiotic prescribing for acute and recurrent cystitis in primary care: a 4 year descriptive study. *Journal of Antimicrobial Chemotherapy*.

[6] Grabe M, Bishop MC, Bjerklund-Johansen TE (2009). *Guidelines on Urological Infections*.

[7] Shaikh N, Morone NE, Bost JE, Farrell MH (2008). Prevalence of urinary tract infection in childhood: a meta-analysis. *Pediatric Infectious Disease Journal*.

[8] Kahlmeter G (2003). An international survey of the antimicrobial susceptibility of pathogens from uncomplicated urinary tract infections: the ECO-SENS project. *Journal of Antimicrobial Chemotherapy*.

[9] Farrell DJ, Morrissey I, de Rubeis D, Robbins M, Felmingham D (2003). A UK multicentre study of the antimicrobial susceptibility of bacterial pathogens causing urinary tract infection. *Journal of Infection*.

[10] Livermore DM, Pearson A (2007). Antibiotic resistance: location, location, location. *Clinical Microbiology and Infection*.

[11] Kass EH (1957). Bacteriuria and the diagnosis of infections of the urinary tract with observations on the use of methionine as a urinary antiseptic. *Archives of Internal Medicine*.

[12] Fahr AM, Eigner U, Armbrust M (2003). Two-center collaborative evaluation of the performance of the BD Phoenix Automated Microbiology System for identification and antimicrobial susceptibility testing of *Enterococcus* spp. and *Staphylococcus* spp. *Journal of Clinical Microbiology*.

[13] De Francesco MA, Ravizzola G, Peroni L, Negrini R, Manca N (2007). Urinary tract infections in Brescia, Italy: etiology of uropathogens and antimicrobial resistance of common uropathogens. *Medical Science Monitor*.

[14] Miragliotta G, Di Pierro MN, Miragliotta L, Mosca A (2008). Antimicrobial resistance among uropathogens responsible for community-acquired urinary tract infections in an Italian community. *Journal of Chemotherapy*.

[15] Daza R, Gutiérrez J, Piédrola G (2001). Antibiotic susceptibility of bacterial strains isolated from patients with community-acquired urinary tract infections. *International Journal of Antimicrobial Agents*.

[16] Schito GC, Naber KG, Botto H (2009). The ARESC study: an international survey on the antimicrobial resistance of pathogens involved in uncomplicated urinary tract infections. *International Journal of Antimicrobial Agents*.

[17] Goldstein FW (2000). Antibiotic susceptibility of bacterial strains isolated from patients with community-acquired urinary tract infections in France. *European Journal of Clinical Microbiology and Infectious Diseases*.

[18] Farrell DJ, Morrissey I, de Rubeis D, Robbins M, Felmingham D (2003). A UK multicentre study of the antimicrobial susceptibility of bacterial pathogens causing urinary tract infection. *Journal of Infection*.

[19] Stratchounski LS, Rafalski VV (2006). Antimicrobial susceptibility of pathogens isolated from adult patients with uncomplicated community-acquired urinary tract infections in the Russian Federation: two multicentre studies, UTIAP-1 and UTIAP-2. *International Journal of Antimicrobial Agents*.

[20] Zhanel GG, Hisanaga TL, Laing NM (2006). Antibiotic resistance in *Escherichia coli* outpatient urinary isolates: final results from the North American Urinary Tract Infection Collaborative Alliance (NAUTICA). *International Journal of Antimicrobial Agents*.

[21] Kiffer CRV, Mendes C, Oplustil CP, Sampaio JL (2007). Antibiotic resistance and trend of urinary pathogens in general outpatients from a major urban city. *International Brazilian Journal of Urology*.

[22] Bergström T (1972). Sex differences in childhood urinary tract infection. *Archives of Disease in Childhood*.

[23] Modarres S, Oskoii NN (1997). Bacterial etiologic agents of urinary tract infection in children in the Islamic Republic of Iran. *Eastern Mediterranean Health Journal*.

[24] Fedler KA, Biedenbach DJ, Jones RN (2006). Assessment of pathogen frequency and resistance patterns among pediatric patient isolates: report from the 2004 SENTRY Antimicrobial Surveillance Program on 3 continents. *Diagnostic Microbiology and Infectious Disease*.

[25] Yüksel S, Oztürk B, Kavaz A (2006). Antibiotic resistance of urinary tract pathogens and evaluation of empirical treatment in Turkish children with urinary tract infections. *International Journal of Antimicrobial Agents*.

[26] Glennon J, Ryan PJ, Keane CT, Rees JPR (1988). Circumcision and periurethral carriage of Proteus mirabilis in boys. *Archives of Disease in Childhood*.

[27] Jaquiery A, Stylianopoulos A, Hogg G, Grover S (1999). Vulvovaginitis: clinical features, aetiology, and microbiology of the genital tract. *Archives of Disease in Childhood*.

[28] Bean DC, Krahe D, Wareham DW (2008). Antimicrobial resistance in community and nosocomial *Escherichia coli* urinary tract isolates, London 2005-2006. *Annals of Clinical Microbiology and Antimicrobials*.

[29] Guneysel O, Onur O, Erdede M, Denizbasi A (2009). Trimethoprim/sulfamethoxazole resistance in urinary tract infections. *Journal of Emergency Medicine*.

[30] Knottnerus BJ, Nys S, Ter Riet G, Donker G, Geerlings SE, Stobberingh E (2008). Fosfomycin tromethamine as second agent for the treatment of acute, uncomplicated urinary tract infections in adult female patients in The Netherlands?. *Journal of Antimicrobial Chemotherapy*.

